# Protective Effect of Field Horsetail Polyphenolic Extract on Erythrocytes and Their Membranes

**DOI:** 10.3390/ijms26073213

**Published:** 2025-03-30

**Authors:** Katarzyna Męczarska, Sylwia Cyboran-Mikołajczyk, Katarzyna Solarska-Ściuk, Jan Oszmiański, Katarzyna Siejak, Dorota Bonarska-Kujawa

**Affiliations:** 1 Department of Physics and Biophysics, Faculty of Biotechnology and Food Sciences, Wrocław University of Environmental and Life Sciences, Norwida 25 St., 50-375 Wrocław, Poland; sylwia.cyboran@upwr.edu.pl (S.C.-M.); 125652@student.upwr.edu.pl (K.S.); 2 Faculty of Biotechnology, Collegium Medicum, University of Rzeszow, Pigonia 1, 35-310 Rzeszow, Poland; 3 Departament of Fruit, Vegetable and Plant Nutraceutical Technology, Faculty of Biotechnology and Food Sciences, Wrocław University of Environmental and Life Sciences, Chełmońskiego 37 St., 51-630 Wrocław, Poland; jan.oszmianski@upwr.edu.pl

**Keywords:** horsetail, plant extracts, antioxidant activity, HMEC-1, transmembrane potential, dipole potential, fluidity, cytotoxicity

## Abstract

Field horsetail (*Equisetum arvense* L.) is widely utilized in traditional medicine and is a rich source of bioactive compounds such as flavonoids, phenolic acids, and silica. This study investigates the protective effect of the polyphenolic extract from field horsetail (HLE) on erythrocytes and their cell membranes. The content of polyphenolic compounds in the extract was determined using the HPLC-DAD and Folin–Ciocalteu methods. The extract’s hemolytic activity, toxicity, antioxidant activity, and its impact on the physical properties of erythrocytes and lipid membrane were investigated. The antioxidant properties were evaluated using erythrocytes and isolated erythrocyte membranes oxidized by UVC radiation and AAPH. The impact of the extract on the ordering and fluidity of erythrocyte and model lipid membranes was studied. Furthermore, the transmembrane potential, shape of erythrocytes and the dipole potential of the lipid membranes under the influence of HLE were evaluated. The results indicated that HLE extract exhibited no toxicity to erythrocytes and HMEC-1 cells. HLE components effectively protect erythrocytes and their membranes against oxidation. They interact with the outer, polar surface of the erythrocyte membrane and reduce both erythrocyte membrane potential and lipid membrane dipole potential. The HLE polyphenols decrease the concentration of free radicals at the surface of the membrane, where they are located, and serve as a protective barrier, preventing penetration into the membrane.

## 1. Introduction

Non-communicable diseases, particularly those affecting the circulatory system, remain some of the most pressing health challenges in modern society. Economic growth and social transformations have led to lifestyle changes that significantly contribute to the increasing prevalence of chronic illnesses [1]. Despite advancements in cardiovascular treatment, mortality rates have not improved substantially, making these diseases the leading cause of death worldwide.

A key factor in the development of circulatory system disorders is oxidative stress, which results from the excessive production of ROS [2]. This process triggers inflammatory responses and damages essential cellular components such as lipids and proteins [3]. One of the earliest indicators of cardiovascular disease is endothelial dysfunction, which plays a crucial role in regulating vascular tone, coagulation, and inflammatory processes. Oxidative stress diminishes the bioavailability of nitric oxide (NO), leading to structural changes in blood vessels that are characteristic of conditions such as hypertension and atherosclerosis [4]. Erythrocytes are particularly vulnerable to oxidative damage, which can degrade membrane proteins (e.g., spectrin and band 3) and reduce the phospholipid content of the membrane [5]. These alterations impair erythrocyte deformability, and prolonged exposure to ROS can ultimately result in hemolysis [6]. Given these risks, there is a growing need to identify natural substances that can help protect against circulatory system diseases and enhance their treatment.

For centuries, plant extracts have been utilized in traditional medicine due to their rich composition of bioactive compounds [7]. Their high content of polyphenols and other phytochemicals endows them with antioxidant, anti-inflammatory, antimicrobial, and anticancer properties. Their beneficial effects, favorable bioavailability, and relatively low toxicity make them a promising subject for further research [8,9,10]. Studies have shown that certain polyphenolic compounds exert a beneficial effect on cardiovascular health. Substances with cardioprotective properties include apigenin, quercetin, and silybin/silymarin, whose effectiveness has been confirmed in both in vitro and in vivo experimental studies. Among the newly identified compounds with protective effects on the cardiovascular system are also naringenin derivatives, such as dihydrochalcone naringin [11,12].

One of the plants widely used in traditional medicine is horsetail, a perennial species recognized for its distinctive morphology and extensive distribution across the northern hemisphere. It thrives in moist soils, particularly in temperate and arctic regions [13,14]. The extract of *E. arvense* has attracted significant interest from fields such as cosmetics, herbal medicine, and pharmacology, prompting increased scientific exploration of its biological properties. This attention is largely due to the plant’s diverse composition, which includes flavonoids, phenolic acids, alkaloids, phytosterols, tannins, saponins, and minerals, particularly silica [15,16].

The potential of horsetail extract extends beyond its traditional uses, as it may enhance conventional therapies by reducing side effects and counteracting drug resistance [17]. Research has demonstrated that horsetail extracts exhibit strong antibacterial activity, effectively inhibiting the growth of pathogens such as *Staphylococcus aureus*, *Escherichia coli*, and *Candida albicans* by disrupting their metabolic processes and inducing cell death [18,19]. Furthermore, the plant’s phytochemical profile contributes to its role in wound healing, anti-inflammatory responses, and bone health. Its high silica content is particularly beneficial for strengthening bones, nails, and connective tissue, making it a popular remedy for osteoporosis and related conditions [20].

In addition, HLE extracts possess significant free-radical-scavenging abilities, suggesting their potential as natural antioxidants [21,22,23]. Despite the numerous studies on horsetail’s medicinal properties, its effects on cardiovascular health remain insufficiently explored. This study aims to investigate the protective mechanisms of polyphenolic extracts from HLE for erythrocytes and their membranes. The research focuses on evaluating the impact of horsetail extract on erythrocyte membrane stability, resistance to oxidative stress, and its possible role in cardiovascular protection.

## 2. Results

### 2.1. Phenolic Content

The horsetail leaf preparation (HLE) was subjected to qualitative and quantitative analysis using chromatographic and mass spectrometric methods. The UV and MS spectra data for HLE extract are shown in the Supplementary Material (Figure S1).

The results, as summarized in Table 1, revealed the presence of a total of 12 low-molecular-weight polyphenolic compounds in HLE. Quercetin derivatives and caffeoyl tartrate isomer derivatives were identified as the major phenolic compounds in the horsetail leaf preparation. Their concentrations were quantified at 135.72 mg/g and 54.0 mg/g of the leaf preparation, respectively. These compounds represent the major phenolic groups within the preparation, underlining their substantial presence and potential contribution to the overall bioactivity of the horsetail extract.

### 2.2. Antiradical Activity

The total polyphenol content (TPC) in the extract was determined using the Folin–Ciocalteu method. The TPC was found to be 197 ± 0.41 mg GAE/g.

The free radical scavenging activity of HLE was assessed based on its ability to reduce the DPPH radical. Within the concentration range of 100–200 µg/mL, the extract exhibited an approximate 10% inhibition of the DPPH radicals (Supplementary Material, Table S1). However, we did not observe a concentration-dependent relationship between DPPH inhibition and HLE concentration.

### 2.3. Interaction of Extract with Erythrocytes and HMEC-1 Cells

**Figure 1:**
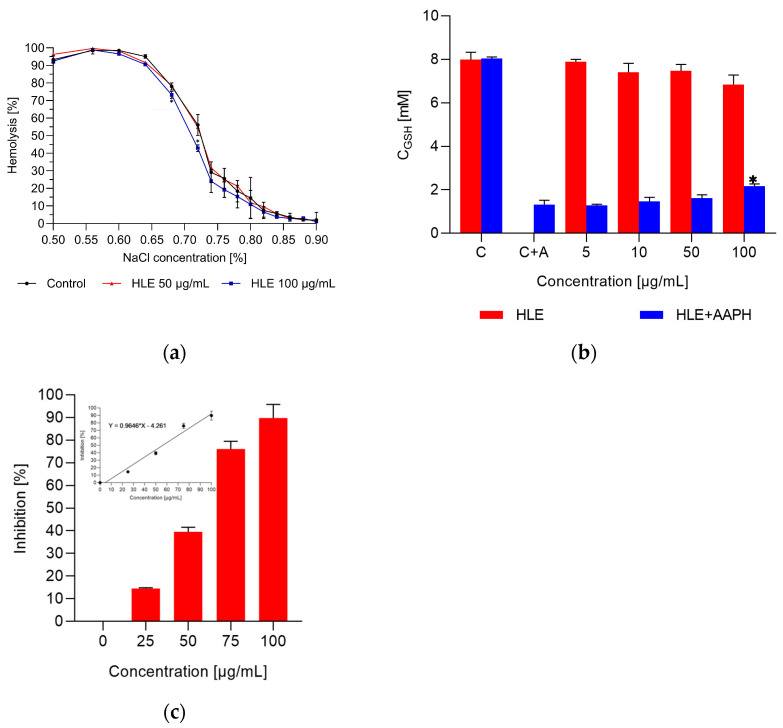
The percentage of hemolysis of erythrocytes modified with 50 µg/mL and 100 µg/mL HLE extract versus NaCl concentration (**a**). Concentration of reduced glutathione (GSH) in red blood cells modified with horsetail leaf extract and exposed to oxidizing agent AAPH (**b**). The percentage of inhibition of erythrocyte oxidation generated by free radicals induced by the AAPH compound (**c**) * Statistically significant results are denoted (α < 0.05).

HLE at concentrations ranging from 0.01 to 0.1 mg/mL did not cause an increase in erythrocyte hemolysis compared with that of the control cells. The extract’s effect on erythrocyte osmotic resistance was assessed by analyzing the hemolysis curves. In the study, no significant differences in hemolysis were observed between the control cells and cells treated with 50 µg/mL of HLE extract at various sodium chloride concentrations (Figure 1a). However, differences were observed between the control cells and cells treated with 100 µg/mL of HLE extract (Figure 1a). The C_50_ values for untreated blood cells and those treated with 100 µg/mL extract were as follows: control C_50_ = 0.72 ± 0.03%; HLE C_50_ = 0.71 ± 0.01%. To confirm the lack of a destructive effect of HLE on the circulatory cells, the extract toxicity was also tested in relation to human vascular endothelial cells (HMEC-1). The concentration of formazan, produced by the reduction of MTT by living cell oxidoreductase enzymes, was used to determine cell viability. After 24 and 72 h of cell incubation with the extract at concentrations between 10 and 200 µg/mL, there was no decreased viability in the extract-treated HMEC-1 cells in comparison to that of unmodified cells (Supplementary Material, Figure S2).

Then, using a scanning electron microscope, the extract’s effect on the shape of the erythrocytes was examined. The images reveal that the extracts induce various echinocyte formations. The results indicate that the polyphenols contained in horsetail extract bind to the red blood cell, resulting in membrane curvature changes (Supplementary Material Figure S3).

In the next study, the effect of HLE on the level of reduced glutathione (GSH) in native erythrocytes and those under oxidative stress induced by AAPH was determined. HLE did not cause a statistically significant change in GSH concentration compared to that of the control erythrocytes (Figure 1b). In red blood cells treated with AAPH, the concentration of reduced glutathione decreased more than fourfold. In the presence of HLE at concentrations ranging from 5 to 50 µg, no changes in GSH levels were observed compared to those for the oxidized red blood cells. Only at the highest concentration used, 100 µg, was a slight increase in GSH levels observed (Figure 1b). The antioxidant activity of the HLE extract was then evaluated based on its ability to inhibit AAPH-induced erythrocyte hemolysis. The results are presented in Figure 1c. The HLE extract protected red blood cells against hemolysis induced by AAPH, with an IC_50_ value of 56.18 ± 0.90 µg/mL. However, its efficacy was approximately two times lower compared to that of the standard antioxidant, ascorbic acid (AA), which had an IC_50_ value of 32.5 ± 4.2 µg/mL.

In the study of the transmembrane potential of the control erythrocytes, when determining the permeability of the cell membrane to potassium ions, a value of −14.73 ± 0.29 was obtained. The modification of erythrocytes with horsetail extract caused a statistically significant decrease in transmembrane potential at both tested concentrations: −16.75 ± 0.38 for 50 µg/mL and −19.86 ± 0.36 for 100 µg/mL. The obtained results suggest that the extract may affect the properties of the erythrocyte membrane by increasing its permeability to potassium ions.

### 2.4. The Impact of the Extract on the Erythrocyte Membrane and Model Lipid Membrane

**Figure 2:**
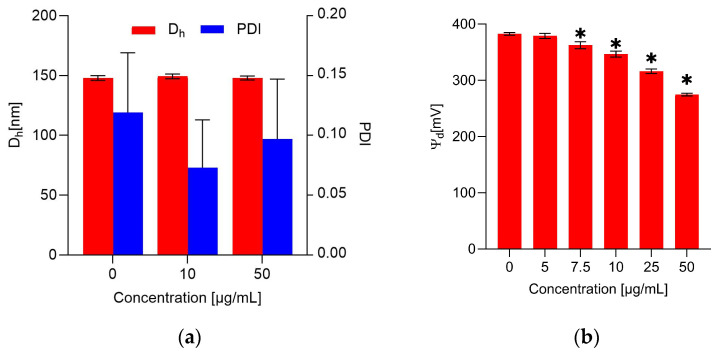
(**a**) The value of the hydrodynamic diameter (D_h_) (nm) and polydispersity index (PDI) of the large monolayer lipid vesicles (LUVs) formed from RBCLs modified with HLE extract, (**b**) changes in dipole potential (Ψ_d_) of liposomes formed from RBCL induced by HLE extract. * Statistically significant results are denoted (α < 0.05).

The DLS study showed that the mean diameter values, D_h_, of the membrane created from lipids extracted from the erythrocyte membrane (RBCL) modified with the HLE extract at concentrations of 10 μg/mL and 50 μg/mL were not significantly different from the diameters obtained for the control unmodified liposomes (Figure 2a). The polydispersity index (PDI) of the control and HLE modified liposomes did not exceed 0.2 (PDI < 0.2). This indicates that the lipid suspension is highly homogeneous.

The impact of HLE on the dipole potential of model lipid membrane was determined by the fluorometric method using the RH-421 probe. The obtained results show that HLE decreased the dipole potential of RBCL in a concentration-dependent manner (Figure 2b).

**Figure 3:**
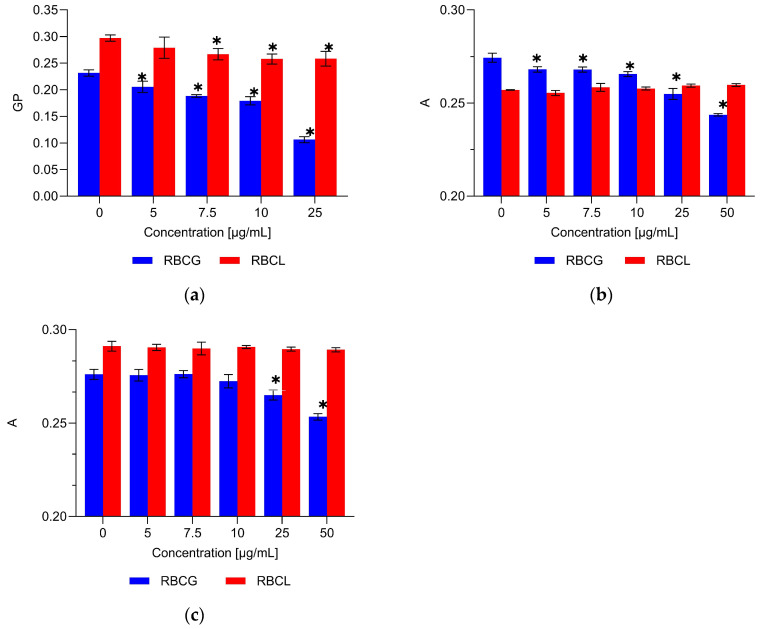
Values of generalized polarization (GP) of the Laurdan probe (**a**), anisotropy (A) of the DPH (**b**), and TMA-DPH probe (**c**) for erythrocytes membrane (ghost, RBCG) and RBCL liposomes modified with the different concentrations (5.0–50.0 µg/mL) of HLE extract at 37 °C. * Statistically significant results are denoted (α < 0.05).

The effect of field horsetail leaf extract on the physical properties of the hydrophilic and hydrophobic regions of erythrocyte membranes (RBCG) and a model membrane formed from lipids extracted from erythrocyte membranes (RBCL) was investigated using DPH, TMA-DPH, and Laurdan fluorescent probes (Figure 3). The membrane fluidity of RBCG and RBCL in the hydrophobic region was determined using DPH and TMA-DPH probes. Significant changes in the fluorescence anisotropy of the DPH and TMA-DPH probes were observed in RBCG membranes treated with HLE extract. No significant changes in fluidity were observed in the RBCL model membranes. Changes in the hydrophilic part of the membrane were determined from the general polarization (GP) of the Laurdan probe. The study showed a significant decrease in the ordering of polar lipid heads in lipid–protein (RBCG) and lipid (RBCL) membranes under the influence of HLE extract. The decrease in GP values suggests that the polyphenolic compounds in the HLE extract bind to the hydrophilic region of the membrane.

### 2.5. Antioxidant Activity of the Extracts

**Figure 4:**
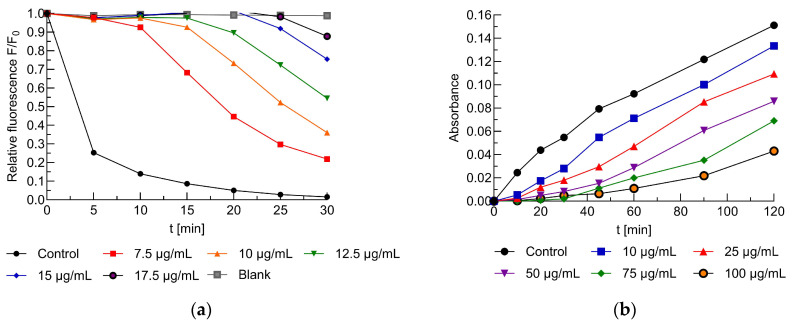
An example of dependencies between (**a**) relative florescence intensity of TMA-DPH probe and time of erythrocyte membrane (RBCG) oxidation induced by AAPH for control and tested samples that contained HLE extract at a concentration range of 7.5–17.5 µg/mL; (**b**) absorbance vs. time of erythrocyte membrane (ghost) oxidation induced by UVC radiation in the presence of HLE extract over a concentration range of 10–100 µg/mL.

The antioxidant activity of horsetail leaf extract (HLE) was evaluated using fluorometric and spectrophotometric methods, with UVC radiation and AAPH as inducers of erythrocyte membrane oxidation. Free radicals generated by AAPH quenched the fluorescence of the TMA-DPH probe, and the relative decrease in fluorescence intensity (F/F₀) was used as an indicator of the degree of lipid oxidation. Figure 4a shows the relationship between relative fluorescence intensity and erythrocyte membrane oxidation time (RBCG) induced by AAPH for the control and HLE-treated samples. Based on the oxidation kinetics, the concentration of the extract required to reduce lipid oxidation by 50% (IC_50_) was determined, which for HLE was 11.8 ± 1.0 µg/mL The results obtained were compared with the antioxidant activity of the following standard antioxidants: ascorbic acid (AA)-20.1 ± 1.10 µg/mL and Trolox^®^-3.90 ± 0.30 µg/mL. The lower IC_50_ value indicates stronger antioxidant properties, suggesting that HLE exhibits significant protective potential against oxidative damage to erythrocyte membranes. Although its antioxidant activity was lower than that of Trolox^®^, it showed greater efficacy than did ascorbic acid.

In the spectrophotometric test, the antioxidant activity of the extracts was evaluated by measuring the concentration of malondialdehyde (MDA) formed during lipid peroxidation. Based on the kinetics of erythrocyte membrane oxidation induced by UVC radiation, the extract concentration required to reduce MDA formation by 50% (IC_50_) was determined. Figure 4b illustrates the representative dependence of absorbance on the oxidation time of erythrocyte membranes induced by UVC (RBCG) in the presence of the HLE extract. As the extract concentration increases, a reduction in MDA formation is observed, confirming its antioxidant activity. The obtained values were as follows: 37.6 ± 1.80 µg/mL for HLE-modified membranes, 16.6 ± 2.60 µg/mL for ascorbic acid (AA), and 14.60 ± 1.30 µg/mL for Trolox^®^.

## 3. Discussion

The primary objective of this study was to investigate the effects of an extract obtained from horsetail (*Equisetum arvense* L.) on red blood cells and their membranes. Polyphenolic compounds were extracted from *HLE*, a species widely distributed across Europe. Chromatographic analysis revealed that the total amount of low-molecular-weight polyphenols in the extract was 219.05 mg/mL. Twelve polyphenolic compounds were identified (Table 1), with quercetin derivatives accounting for over 61% of the bioactive substances in the extract. Quercetin-3-O-6-acetylglucoside, an isomer of quercetin glucosides, has been shown to exhibit greater bioavailability than quercetin itself, thereby enhancing its therapeutic potential. These compounds possess a broad spectrum of pharmacological properties, including antioxidant, anti-proliferative, anti-inflammatory, antihypertensive, and anti-diabetic activities [24,25,26]. The second most abundant compound in the studied extract was a caffeoyl tartrate isomer, contributing approximately 25% of the total polyphenols. Different isomeric forms of this compound exhibit diverse biological properties, making it relevant for both medicinal and food applications [27]. Other identified fractions included phenolic acids (approximately 10%) and kaempferol derivatives (2%). The obtained results align with those in the existing literature regarding the composition of horsetail extracts [13,16,28,29]. Studies by Veit et al. have shown that the major constituent of *Equisetum arvense* L. is quercetin 3-O-glucoside, which is present in significant amounts, along with other glycosides, such as kaempferol 3-O-glucoside and apigenin 5-O-glucoside [30]. Similarly, Gazafroudi et al. [29] confirmed that the predominant class of secondary metabolites in horsetail extracts consists of polyphenolic glycosides, including derivatives of myricetin, quercetin, and kaempferol. In addition, *E. arvense* L. extract is characterized by the presence of acetyl conjugated glycosides. Our analysis confirmed these findings [29] and “revealed the generation of [aglycone-H] ions at *m*/*z* 285 and 301, suggesting the presence of kaempferol and quercetin acetylated glycosides” [29]. In addition, studies indicate the presence of derivatives and isomers of hydroxycinnamic acid, such as caffeoylquinic acid, in horsetail extract. [16,29].

The toxicity of horsetail extract to normal HMEC-1 cells and erythrocytes has not been directly addressed in the literature. Therefore, this study aimed to determine whether the extract exhibits hemolytic activity against red blood cells. The results indicated that the HLE extract was not destructive to erythrocytes at concentrations ranging from 10 to 100 µg/mL. An MTT assay on immortalized human microvascular endothelial cells (HMEC-1) (Supplementary Material, Figure S2) also confirmed the absence of toxic effects of HLE.

The lack of hemolytic activity allowed for further investigations regarding the impact of HLE on the physical properties of erythrocytes and their membranes. The osmotic resistance of erythrocytes is a key parameter that determines the integrity and stability of the cell membrane. Osmotic resistance analysis provides important information about the health and function of erythrocytes under various conditions. Therefore, in the next step, the osmotic resistance of erythrocytes exposed to HLE was examined. These studies showed that HLE does not change the osmotic resistance of erythrocytes (Figure 1a). Furthermore, scanning electron microscopy revealed a shape change of the cells treated with the HLE from discocytes to echinocytes (Figure 1c). The shape change of red blood cells suggests that compounds contained in HLE bind to the membrane, primarily in the outer layer of the lipid bilayer.

The next important parameter of biological membranes comprises membrane potential, especially transmembrane potential, whose alterations may affect the shape of erythrocytes and the formation of echinocytes [31]. The transmembrane potential of erythrocytes treated with HLE extract exhibit a lower negative value compared to that of the control erythrocytes. This is linked to an increase in the extra-cellular potassium ion concentration, which plays a role in the distribution of membrane proteins and lipids, potentially affecting membrane function [32].

Studies of the effect of HLE extract on intact erythrocytes indicate that the extract primarily localizes in the outer region of the cell membrane. This is evidenced by the transformation of erythrocytes into echinocytes and the reduction in transmembrane potential. To confirm these observations, the concentration of reduced glutathione (GSH) was measured both during incubation with HLE and under oxidative stress conditions induced by AAPH. The GSH levels in erythrocytes play a crucial role in membrane stabilization, improving cellular redox status by neutralizing free radicals, and reducing the risk of lipid peroxidation, which compromises membrane structure. Consequently, the membrane maintains its integrity and flexibility. Additionally, GSH prevents the oxidation and aggregation of membrane proteins, which is essential for the proper functioning of membrane transporters and the overall stability of the cell [33,34]. In our study, HLE extract did not affect GSH levels compared to those of the control cells. However, in erythrocytes exposed to AAPH, an increase in GSH levels was observed exclusively in cells treated with 100 µM of HLE extract compared to those of the AAPH-treated erythrocytes. The extract likely exerts its effect by capturing and neutralizing free radicals near the membrane, thereby reducing oxidative stress and increasing GSH availability in erythrocytes. This assumption is further supported by the AAPH-induced hemolysis assay, which demonstrated that HLE exerts a protective effect on erythrocytes by reducing hemolysis associated with oxidative stress. In order to determine the molecular mechanism of HLE interaction with erythrocytes, additional studies were conducted on biological and model membranes. Firstly, to assess the impact of HLE on membrane dipole potential, a voltage-sensitive probe, RH 421, was used. RH 421 was incorporated into an RBCL membrane. This probe exhibits changes in fluorescence lifetime and intensity due to the internal electric field of the membrane [35]. It was found that the HLE extract decreases the R ratio of the RH 421 probe, which is proportional to the dipole potential, thus reducing it. These results confirmed previous findings regarding the transmembrane potential of erythrocytes and indicate that the HLE extract alters the charge distribution within the membrane. This effect may result from the interaction of HLE with both the hydrophilic and hydrophobic regions of the membrane.

To verify the localization, studies were conducted to examine the effect of the extract on the physical properties of erythrocyte membranes and lipid model membranes. Laurdan fluorescent probes, DPH, and TMA-DPH were used for this purpose [36,37]. The studies showed that the compounds contained in HLE do not affect the anisotropy of DPH and TMA-DPH probes in the hydrophobic region of the lipid membrane but do reduce the GP of the Laurdan probe. These results indicate that the extract’s components are mainly localized in the hydrophilic region of the lipid membrane and do not penetrate its core. In the case of the lipid–protein erythrocyte membrane, small changes in DPH anisotropy were observed. Changes in TMA-DPH anisotropy were noted only at the highest concentrations used. In the lipid head group region, a concentration-dependent drop in the GP values of the Laurdan probe was observed. This means that HLE components bind to the polar heads of lipids, causing an increase in their disorder. The observed changes in the hydrocarbon chains are likely due to alterations in the hydrophilic region of the membrane.

These results also indicate that HLE induces greater changes in the lipid–protein membrane than in the lipid membrane alone, suggesting that the extract interacts not only with lipids but also with membrane proteins. The interaction of HLE compounds with the hydrophilic region of the membrane is consistent with scientific reports stating that quercetin and its derivatives, which are major components of the extract, bind primarily to the membrane surface. Quercetin forms hydrogen bonds with the polar head groups of the lipid bilayer, preventing its entry into the hydrophobic core due to a high energetic barrier. This influences the distribution of quercetin and its antioxidant properties [38,39]. The HLE extract, rich in quercetin derivatives, localizes on the membrane surface, potentially enhancing its resistance to oxidative damage. Studies by Pawlikowska-Pawłęga et al. (2007) confirm the localization of quercetin in the polar head group region at physiological pH [40]. They have also shown that quercetin modifies the erythrocyte membrane through interactions with cytoskeletal proteins [41]. Like quercetin glycosides, phenolic acids tend to localize at the membrane surface due to their hydrophilic nature, primarily through hydrogen bonding. These interactions can modify membrane properties, enhancing stability and reducing permeability to harmful molecules, thereby contributing to their antioxidant effects [42,43,44,45].

The antioxidant activity of horsetail leaf extract was evaluated using fluorometric and spectrophotometric methods. In the studies, UVC radiation and AAPH were used as oxidative stress inducers for erythrocyte membranes. Based on oxidation kinetics, the IC_50_ values were determined (the concentration required to reduce oxidation by 50%) for the extract, as well as for the standard antioxidants AA and Trolox^®^. The HLE extract exhibited stronger protective properties against oxidation induced by AAPH compared to UVC radiation-induced free radical damage. In AAPH-induced oxidation, HLE extract showed slightly lower antioxidant activity than did Trolox^®^, but it was nearly twice as high as that of AA. The studies demonstrated that extracts from *Equisetum arvense* L. have the ability to reduce lipid peroxidation and alleviate oxidative stress in cells due to their potent antioxidant properties. They contain a rich array of phenolic and flavonoid compounds that play a key role in neutralizing free radicals and protecting cells from oxidative damage. This effect depends on the dose and varies according to the type of extract used [29,46,47].

The compounds present in field horsetail extract may protect cells from oxidative stress, inflammation, toxins, and metabolic damage. One of the key substances found in horsetail are flavonoids, including quercetin derivatives, particularly its acetylglucosylated forms, which exhibit strong antioxidant properties. As a result, they can neutralize reactive oxygen species (ROS) and protect cells from oxidative stress. According to Abdel-Daim et al., flavonoids may display cardioprotective effects, reducing inflammation, improving endothelial function, and shielding the circulatory system from oxidative damage [48]. Another significant group of compounds includes phenolic acids, which demonstrate anti-inflammatory and antioxidant properties and may support tissue regeneration. Polyphenolic compounds present in horsetail, like other antioxidants, help reduce the oxidative damage of lipids, which may contribute to lowering the risk of chronic diseases [48,49]. However, as noted by Pallag et al., the antioxidant activity of field horsetail depends on climatic conditions [50]. There is a strong correlation between the accumulation of antioxidant compounds in the plant and the environmental conditions in which it grows. This has significant implications for both the therapeutic efficacy of HLE and sustainable harvesting practices, as well as the potential development of plant-based medicines [50]. In conclusion, the compounds found in field horsetail may promote health by protecting organism against oxidative stress. While further research is needed to explore their full potential in regards to disease prevention and treatment, their beneficial impact on the cardiovascular system and overall homeostasis is already being recognized.

One limitation of this study is it’s in vitro nature. Due to the complexity of processes occurring in the human body, the results can only suggest the potential effects of the examined extract in vivo. Nevertheless, the study shows several significant strengths. The experimental models included both lipids extracted from erythrocytes and erythrocyte ghosts, allowing for the analysis of interactions in both the lipid phase and the protein–lipid phase. Another important aspect is the use of UVC radiation as an oxidative factor; most studies focus on chemical oxidants, which makes this research unique and broadens the scientific perspective.

## 4. Materials and Methods

### 4.1. Materials

#### 4.1.1. Reagents

The fluorescent probes 6-dodecanoyl-2-dimethylaminonaphthalene (Laurdan), CAS No.: 74515-25-6; 1,6-diphenyl-1,3,5-hexatriene (DPH), CAS No.: 484049-04-9; 1-(4-trimethylammoniumphenyl)-6-phenyl-1,3,5-hexatrienep-toluenesulfonate (TMA-DPH), CAS No.: 115534-33-3; N- (4-sulfobutyl)-4-(4-(4-(dipentylamino) phenyl) butadienyl) pyridinium (RH421), CAS No.: 381236-90-4; 3,30-dipropylthiadicarbocyanine iodide (DiSC3(5)), CAS No.: 53213-94-8; 2,2-diphenyl-1-picrylhydrazyl (DPPH), CAS No.: 1898-66-4; the 2,2′-azobis(2-methylpropionamidine) dihydrochloride (AAPH), CAS No.: 2997-92-4; Follin–Ciocâlteu reagent, CAS No.: 1898-66-4; glutar aldehyde, 5,5′-dithio-bis-(2-nitrobenzoic acid) (DTNB), CAS No.: 69-78-3 were purchased from Merck (Rahway, NJ, USA). Heparin was obtained from Polfa Warszawa (Warszawa, Poland). Substances used for the preparation of buffers included NaCl (Avantor Performance Materials, Gliwice, Poland), Na_2_HPO_4_·H_2_O, NaH_2_PO_4_·H_2_O, EDTA, and Tris (Chempur, Piekary Śląskie, Poland). Ethanol was purchased from Avantor Performance Materials, Gliwice, Poland. Thiobarbituric acid (TBA) and immersion oil were obtained from Honeywell Fluka (Charlotte, NC, USA), and trichloroacetic acid (TCA) was purchased from Eurochem BGD (Tarnów, Poland).

#### 4.1.2. Plant Material and Extraction Procedure

The horsetail (*Equisetum arvense* L.) leaves were harvested from the Medicinal Plant Garden of Wrocław Medical University, Poland. After collection, they were immediately flash-frozen in liquid nitrogen and lyophilized (24 h; Alpha 1–4 LSC, Christ, Osterode am Harz, Germany). Homogeneous powders were obtained by grinding the dried tissues in a sealed laboratory mill to prevent moisture absorption. The powders were stored at −80 °C until extract preparation. The extract of horsetail leaves was provided by the Department of Fruit, Vegetable, and Plant Nutraceutical Technology at the University of Environmental and Life Sciences in Wroclaw. The polyphenols were extracted from the leaves using a method previously described by Gąsiorowski et al. [51]. The extraction was performed with water containing 200 ppm SO_2_, using a solvent/material ratio of 3:1 (*v*/*v*). The extract was then purified by adsorption on Purolite AP 400 resin, followed by elution with 80% ethanol. The polyphenols were then concentrated and lyophilized.

#### 4.1.3. Red Blood Cells, HMEC-1 Cells, and Model Membranes

In the studies, the erythrocytes isolated from fresh heparinized porcine blood, together with an immortalized human skin microvascular endothelial cell line, HMEC-1, were used. The erythrocytes were prepared according to a previously established method [52]. The HMEC-1 cell line (ATCC CRL 3243) was obtained from the American Type Culture Collection (ATCC, Dziekanów Leśny, Poland), and cell culture was performed according to standard procedures briefly described in. Solarska-ściuk et al. [52]. Porcine blood was obtained from a slaughterhouse. According to Polish regulations, ethical committee approval was not required for its use in these experiments.

Erythrocyte membranes (RBCG) were prepared from fresh blood using the method previously described by Dodge [53]. The protein concentration of the sample was determined by the Lowry method [54] and was approximately 100 μg/mL.

Lipids were extracted from the erythrocytes (RBCL) to a final concentration of 10 mg/mL using the method described by Maddy [55]. To prepare large unilamellar vesicle LUVs, the lipid was evaporated in a stream of liquid nitrogen, followed by drying in a vacuum evaporator. The resulting lipid film was then hydrated in a buffer suitable for the assay procedure, followed by vortex shaking for several minutes. The resulting MLV liposomes were extruded through 200 nm and 100 nm polycarbonate filters from Whatman^®^ Nuclepore TM, GE Healthcare, Chicago, IL, USA, using a Lipex automatic pressure extruder from Evonik Industries AG, Essen, Germany.

### 4.2. Methods

#### 4.2.1. Phenolic Content

##### UPLC-DAD, UPLC–ESI–MS

The preparation of horsetail leaf extract was analyzed using UPLC–ESI–MS–MS systems. Qualitative analysis was performed using LC-DAD–MS-MS methods, and quantitative analysis was performed using UPLC-MS (quantified by both DAD and MS detection), as described by Oszmiański et al. [56].

#### 4.2.2. Antiradical Activity

Total phenolic content was determined using Folin–Ciocalteu reagent, according to the method of Singleton et al. [57]. A standard curve was constructed using gallic acid. The results were expressed as milligrams of gallic acid equivalents (GAE) per gram of dry sample.

The determination of the antiradical activity was performed using the DPPH method, based on the method described by Kaźmierczak et al. [11]

#### 4.2.3. Interaction of Extract with Erythrocytes and HMEC-1 Cells

##### Hemolysis and Osmotic Resistance of Erythrocytes

The hemolytic activity of the extract and its impact on the osmotic resistance of erythrocytes were determined using the method described previously [4], with minor modifications. The erythrocyte suspension with a hematocrit of 1.2% was incubated for 1 h at 37 °C with the extract at a concentration of up to 100 µg/mL. The hemoglobin concentration in the supernatant was then measured at a wavelength of 540 nm. In the osmotic resistance study, red blood cells with a hematocrit of 1.2% were incubated for 1 h at 37 °C with HLE extract at a concentration of 50 µg/mL and 100 µg/mL. The samples were then centrifuged and the supernatant removed. The erythrocytes modified with the extract were suspended in NaCl containing tubes at concentrations ranging from 0.50% to 0.9%. The measurement was performed spectrophotometrically at a wavelength of 540 after one hour of incubation. A SPECORD^®^ 40 spectrophotometer (Analytik Jena AG, Jena, Germany) was used for the measurements.

##### MTT Cytotoxicity Assay

The effect of the extract on the viability of HMEC-1 cells was assessed using the MTT (tetrazole dye 3-(4,5-dimethylthiazol-2-yl)-2,5-diphenyltetrazolium bromide) method [52]. Cells were seeded in 96-well plates at 5000 cells per well and cultured for 12–24 h. HLE extract was added at concentrations ranging from 0 to 200 μg/mL and incubated for 24, 48, and 72 h. After incubation, cell monolayers were washed with Hanks’ salt solution (HBSS), and then fresh medium was added, along with 20 μL of 5 mg/mL MTT solution. After 2 h of incubation, the medium was removed, and the formazan crystals were dissolved with DMSO. The absorbance of formazan was measured at 570 nm on the Agilent BioTek Epoch Microplate Spectrophotometer (Agilent Technologies, Santa Clara, CA, USA).

##### Concentrations of Reduced Glutathione in Red Blood Cells Exposed to Free Radicals

A method for determining the concentration of the reduced form of glutathione (GSH) has been described in the literature by Ellman [53]. A total of 2 mL of porcine blood with a hematocrit of 10% was incubated for 1 h with HLE extract at concentrations of 5 μg/mL, 10 μg/mL, 50 μg/mL, and 100 μg/mL. After incubation, AAPH was added to the samples to bring the concentration in the sample to 60 mM, and the sample was incubated for an additional hour. Then, 200 μL of cold TCA 25% was added and centrifuged at 3500 rpm for 10 min at 4 °C. After centrifugation, 1 mL of supernatant was collected, and 1 mL of 0.5 M phosphate buffer pH = 8.2 was added. A total of 100 µL of DTNB reagent with a starting concentration of 5 mM was then added and incubated for 30 min in the dark at room temperature. The absorbance A of the samples at 412 nm, relative to the reference sample, was measured using a SPECORD^®^ 40 (Analytik Jena AG, Jena, Germany) spectrophotometer. The mean concentration of TNB (5-thio-2-nitrobenzoate anion) was calculated using Equation (1), where *ε* is the molar absorption coefficient of TNB, which is 1360 dm^3^mol^−1^cm^−1^, and the hematocrit is the optical path length.

(1) $$C_{TNB}=\frac{A}{\varepsilon l}$$

In the next step, the GSH content of the control and HLE extract-modified erythrocytes was calculated using Equation (2), where *H* is the hematocrit.

(2) $$C_{GSH}=\frac{C_{TNB}\cdot 2.1\cdot 1.1}{H}\cdot 100\%$$

##### Protection Against Free-Radical-Induced Hemolysis

The method assessing the protection against free-radical-induced hemolysis was performed based on a method previously described by us [58]. A SPECORD^®^ 40 spectrophotometer (Analytik Jena AG, Jena, Germany) was used for the measurements.

##### Fluorometric Determination of Erythrocyte Transmembrane Potential

The transmembrane potential of the erythrocytes was determined based on the method described by Cyboran-Mikołajczyk et al. [4], with slight modifications. A total of 1 mL of erythrocyte suspension with a hematocrit of 5% was incubated for one hour with HLE extract at concentrations of 50 μg/mL and 100 μg/mL. Then, 100 μL of blood was suspended in a buffered physiological saline solution containing 10 mM Tris-HCl (pH 7.4) and 150 mM (KCl + NaCl), with the K+ concentration gradually increased from 10 to 140 mM. The prepared sample was incubated at room temperature for 10 min. In the next step, the DiSC3(5) probe was added to the sample at a final concentration of 2 µM, followed by incubation for 45 min in the dark at room temperature. The fluorescence intensity of the dye (I) was measured using a CARY Eclipse fluorimeter (Varian, Plato Alto, CA, USA) at an emission wavelength of 660 nm and an excitation wavelength of 625 nm. Valinomycin was then added to the sample at a final concentration of 1 µM, followed by incubation for 10 min, after which the fluorescence intensity of the probe (I_v_) was measured again.

##### Microscopic Studies of Erythrocyte Shapes

The effect of the extract on erythrocyte morphology was assessed using scanning electron microscopy (SEM), following a previously described method developed by our co-workers, with slight modifications [4]. For SEM microscopy analysis, the HLE extract was applied at a concentration of 50 μg/mL. The ultrastructure of the material was examined using a scanning electron microscope (EVO LS15 ZEISS, Oberkochen, Germany) with an SE1 detector, operating under high vacuum and an accelerating voltage of 20 kV.

#### 4.2.4. The Impact of the Extract on the Erythrocyte Membrane and Model Lipid Membrane

##### Determination of the Hydrodynamic Diameter of Liposomes and the Polydispersity Index

The hydrodynamic diameter (D_h_) and polydispersity index (PDI) of the HLE-modified LUVs were determined using a nanoPartica SZ-100Vs laser particle size analyzer (Horiba Ltd., Minamiku, Kyoto, Japan). The method was previously described by Cyboran- Mikołajczyk et al. [59], with minor modifications. The concentrations of horsetail leaf extract in the suspension of liposomes formed from lipids extracted from RBCL were 10 μg/mL and 50 μg/mL, respectively, measured after 45 min of incubation at 25 °C.

##### Fluorometric Dipole Potential Measurement

The dipole potential of lipid membranes formed from lipids extracted from erythrocyte membrane was determined by a fluorometric method using the RH 421 probe described earlier by Cyboran-Mikołajczyk et al. [59]. Extract concentrations in the samples ranged from 5 to 50 μg/mL. The molar ratio of the concentration of lipid to fluorescent probe was 100:1. The excitation spectra of the probe were recorded in the 400–600 nm range at a temperature of 23 °C, using an emission wavelength of 650 nm. A Cary Eclipse spectrophotometer (Varian, Plato Alto, CA, USA) was used for the measurements, with both the excitation and emission slits set to 5 nm.

##### Packing Order and Fluidity of the Membrane

A fluorometric method described earlier by our co-workers, with slight modifications [48], was used to investigate the effect of the HLE on the packing order and fluidity of erythrocyte membrane (ghost) and model lipid (RBCL) membranes. The fluorescence intensity of the DPH, TMA-DPH, and Laurdan probes was measured at 37 °C. The extract concentration in the sample ranged from 5 to 50 μg/mL. The molar ratio of the concentration of lipid to fluorescent probe was 100:1. The fluorescence anisotropy of the DPH and TMA-DPH probes and the generalized polarization GP values of the Laurdan probe were calculated according to the method described by Parasassi et al. [60] and Lakowicz [61]. A Cary Eclipse spectrophotometer (Varian, Plato alto, CA, USA USA) was used for the measurements, with both the excitation and emission slits set to 5 nm.

#### 4.2.5. Antioxidant Activity of the Extracts

The antioxidant activity of the HLE extract against the erythrocyte membranes was determined spectrophotometrically and fluorometrically using two oxidation inducers, UVC and AAPH, according the method described earlier by Singleton et al. [57]. The antioxidant activity was determined on the basis of the IC_50_ concentration responsible for a 50% inhibition of lipid peroxidation. The antioxidant activity was compared with that of standard antioxidants such as Trolox^®^ and L(+) ascorbic acid (AA). A Cary Eclipse spectrophotometer (Varian, Plato alto, CA, USA) and a SPECORD^®^ 40 spectrophotometer (Analytik Jena AG, Jena, Germany) were used for the measurements.

## 5. Conclusions

The polyphenolic compounds in horsetail extract, primarily quercetin derivatives and phenolic acids, do not exhibit cytotoxic effects on erythrocytes or HMEC-1 cells. These compounds predominantly localize in the hydrophilic regions of erythrocyte membranes, influencing membrane organization, fluidity, and dipole potential. This localization likely underlies the extract’s protective effects by reducing free radical concentrations near the membrane and enhancing its antioxidant capacity. Given these properties, horsetail extract holds promise for its incorporation into dietary supplements and functional foods to support cardiovascular health. However, further comprehensive trials are essential to confirm its efficacy and long-term safety.

## Figures and Tables

**Table 1:** Identification and content of phenolic acids and flavonoids in HLE leaf extract. Retention time (R_t_),wavelengths of maximum absorption in the UV–visible region (λ_max_), and mass spectral data (molecular ions and MS_2_).

Compounds	Content mg/g	R_t_ min	λ_max_ nm	Molecular ion [M-H]^−^ *m*/*z*	MS_2_ *m*/*z*
Caffeoyl tartrate isomer	3.66	3.47	327	311	179
Caffeoyl tartrate isomer	50.34	3.57	327	311	179
Caffeoylshikimic acid isomer Kaempherol diglycoside	3.81	4.96	327	335	179
Quercetin dihexoside	4.91	5.03	342	609	285
Quercetin-glucoside	24.88	5.76	350	625	301
Quercetin-3-O-6-acetylglucoside isomer	22.89	7.32	351	463	301
Quercetin-3-O-6-acetylglucoside isomer	81.58	7.95	353	505	301
Quercetin-3-O-6-acetylglucoside isomer	3.83	8.32	353	505	301
Kaempferol-3-O-6-acetylglucoside	1.23	8.96	353	505	301
Dicaffeoyl tartaric acid isomer	1.31	9.32	340	489	285
Dicaffeoyl tartaric acid isomer	15.92	9.55	328	473	179
Total	219.05				

## Data Availability

The data presented in this study are available in this article and Supplementary Materials, available online: https://bazawiedzy.upwr.edu.pl/info/researchdata/UPWR821989684bb54f09a20134d315c371db/, DOI:10.57755/w2qe-w396, accessed 28 March 2025.

## Supplementary Materials

The following supporting information can be downloaded at: https://www.mdpi.com/article/10.3390/ijms26073213/s1.
