# Supplementary material for: Protective Effect of Field Horsetail Polyphenolic Extract on Erythrocytes and Their Membranes
Source: Int J Mol Sci. 2025 Mar 30;26(7):3213. doi: 10.3390/ijms26073213 (PMC11989917; doi:10.3390/ijms26073213)
Supplement: Supplementary file 1 [file ijms-26-03213-s001.zip › ijms-3519527-supplementary.pdf]

Protective Effect of Field Horsetail Polyphenolic Extract on Erythrocytes and Their Membranes

Katarzyna Męczarska [1,*], Sylwia Cyboran-Mikołajczyk [1], Katarzyna Solarska-Ściuk [2], Jan Oszmiański [3], Katarzyna Siejak [1] and Dorota Bonarska-Kujawa [1,*]

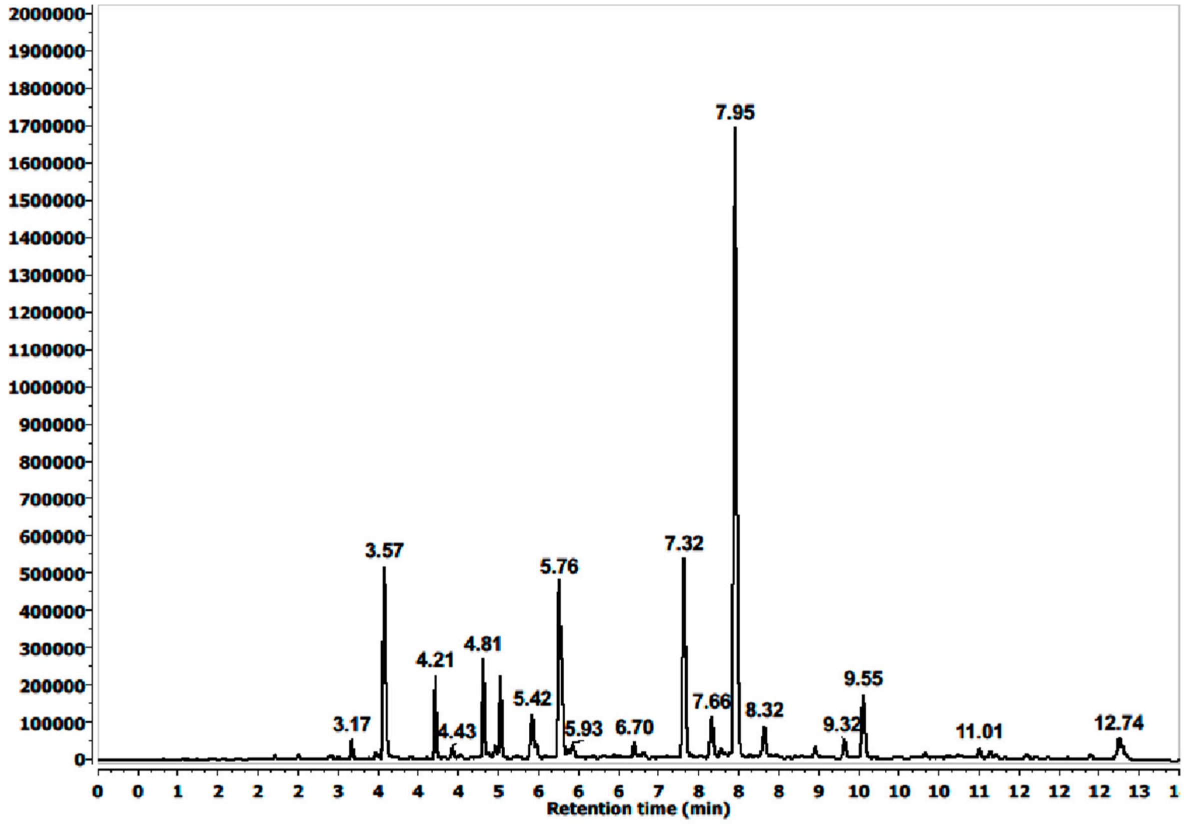

Figure S1. HPLC Chromatogram at 360 nm of flavonoids in *Equisetum arvense* L. (common horsetail) leaf extracts.

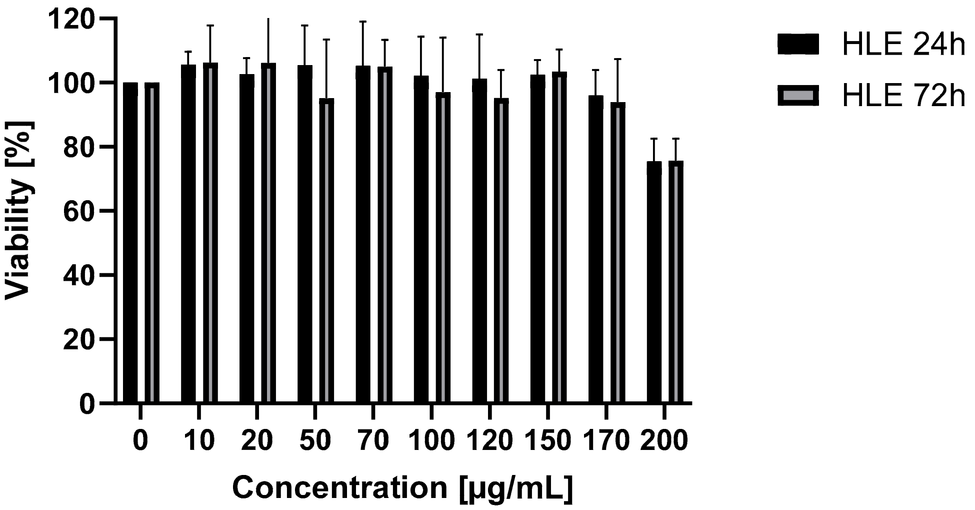

Figure S2. Cytotoxicity of HLE extract in relation to HMEC-1 cells. Cell viability of human dermal microvascular endothelial cells line of HLE was determined by MTT assay after 24 h and 72 h exposure.

Table S1. Inhibition of DPPH radicals (%).

Concentration [µg/mL]	I	II	III	Averge	SD
**0**	**0,00**	**0,00**	**0,00**	**0,00**	0
**25**	**19,11**	**10,91**	**0,00**	**10,00**	9,584648
**50**	**5,87**	**1,41**	**0,00**	**0,00**	3,062715
**75**	**8,26**	**4,18**	**0,00**	**4,15**	4,130197
**100**	**5,35**	**17,46**	**4,10**	**8,97**	7,379387
**125**	**8,55**	**4,11**	**10,21**	**7,62**	3,153205
**150**	**10,98**	**18,10**	**9,23**	**12,77**	4,698848
**175**	**13,95**	**9,22**	**0,00**	**7,72**	7,093312
**200**	**18,01**	**14,54**	**10,03**	**14,19**	3,999917

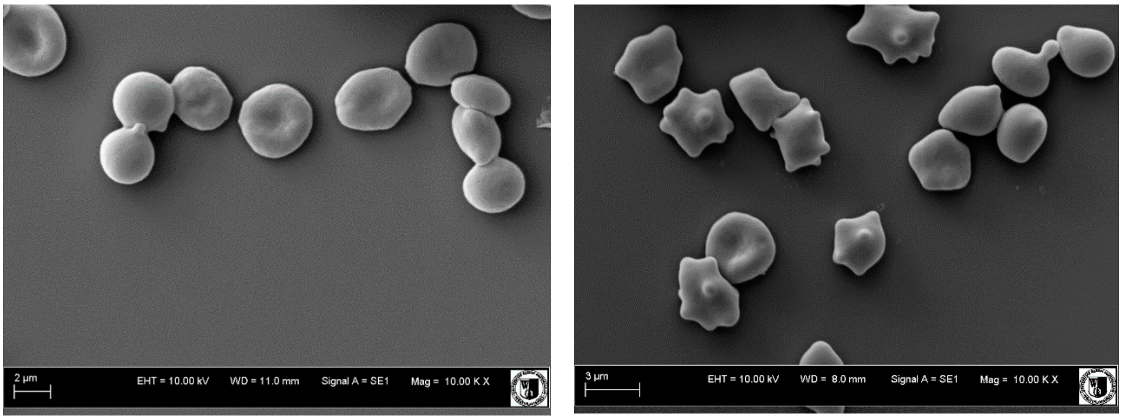

(a) (b)

Figure S3. Shapes of erythrocytes observed under an electron scanning microscope, including unmodified cells (a) and treated with HLE at a concentration of 50 µg/mL (b).
